# Neuroimaging in repetitive brain trauma

**DOI:** 10.1186/alzrt239

**Published:** 2014-02-24

**Authors:** Thomas SC Ng, Alexander P Lin, Inga K Koerte, Ofer Pasternak, Huijun Liao, Sai Merugumala, Sylvain Bouix, Martha E Shenton

**Affiliations:** 1 Center for Clinical Spectroscopy, Department of Radiology, Brigham and Women’s Hospital, Harvard Medical School, 4 Blackfan Circle, Boston, MA 02115, USA; 2 Keck School of Medicine of the University of Southern California, 1975 Zonal Ave, Los Angeles, CA 90033, USA; 3 Psychiatric Neuroimaging Laboratory, Departments of Psychiatry and Radiology, Brigham and Women’s Hospital, Harvard Medical School, 1249 Boylston Street, Boston, MA 02215, USA; 4 Institute for Clinical Radiology, Ludwig-Maximilians-University, Marchioninistrasse 15, 81377 Munich, Germany; 5 Research and Development, VA Boston Healthcare System, 850 Belmont Street, Brockton, MA 02130, USA

## Abstract

Sports-related concussions are one of the major causes of mild traumatic brain injury. Although most patients recover completely within days to weeks, those who experience repetitive brain trauma (RBT) may be at risk for developing a condition known as chronic traumatic encephalopathy (CTE). While this condition is most commonly observed in athletes who experience repetitive concussive and/or subconcussive blows to the head, such as boxers, football players, or hockey players, CTE may also affect soldiers on active duty. Currently, the only means by which to diagnose CTE is by the presence of phosphorylated tau aggregations post-mortem. Non-invasive neuroimaging, however, may allow early diagnosis as well as improve our understanding of the underlying pathophysiology of RBT. The purpose of this article is to review advanced neuroimaging methods used to investigate RBT, including diffusion tensor imaging, magnetic resonance spectroscopy, functional magnetic resonance imaging, susceptibility weighted imaging, and positron emission tomography. While there is a considerable literature using these methods in brain injury in general, the focus of this review is on RBT and those subject populations currently known to be susceptible to RBT, namely athletes and soldiers. Further, while direct detection of CTE *in vivo* has not yet been achieved, all of the methods described in this review provide insight into RBT and will likely lead to a better characterization (diagnosis), *in vivo*, of CTE than measures of self-report.

## Introduction

Between the years 2000 and 2012, over 266,810 service members sustained at least one concussion [1]. Furthermore, 1.6 to 3.8 million individuals in the United States experience a sports-related concussion [2] every year, with a growing number of these events in youth sports participants [3]. The incidence of repetitive subconcussive blows (that is, hits to the head with enough force to hamper neuronal integrity, but without associated symptoms) is thought to be much greater [4]. For example, Broglio and colleagues [5] have found that high school football players receive an average of 652 hits to the head per season that exceed 15 Gs of force.

The pathological effects of repetitive brain trauma (RBT) are thus a growing concern, especially with the discovery of chronic traumatic encephalopathy (CTE), a neurodegenerative disease marked by widespread accumulation of hyperphosphorylated tau, predominantly present as neurofibrillary and astrocytic tangles as witnessed in post-mortem brains [6,7]. CTE has been found most often in professional athletes involved in contact sports (for example, boxing, American football) who have been subjected to RBT, including mild traumatic brain injury (mTBI; or concussion) or even asymptomatic, subconcussive trauma. Neuropathologically confirmed CTE has been reported in individuals aged as young as 17 years and in contact sport athletes who played competitive sports only through high school or college. Moreover, CTE has been found in non-athletes who have experienced RBT, including individuals with epilepsy, developmentally disabled individuals with head banging, and victims of physical abuse [6,8]. Recently, CTE has been neuropathologically diagnosed in soldiers with histories of RBT deployed in Iraq and Afghanistan [6,9]. All cases of neuropathologically confirmed CTE reported to date have had a history of RBT, indicating that RBT may be a necessary variable for the initiation of the pathogenetic cascade that eventually leads to neurodegeneration.

Symptoms of neurodegeneration begin years or decades after RBT exposure and include changes in cognition, mood, and behavior [10]. As the disease progresses, it can lead to dementia. The incidence and prevalence of CTE are unknown, though the number of those affected is potentially quite large. With over one million US high school students playing football each year, the public health impact of RBT, in general, and the development of CTE, in particular, is quite significant.

It is important to note that, while RBT may be a necessary condition for developing CTE, the mechanism by which RBT may lead to CTE is unknown. Further research about the pathophysiological pathway that leads to CTE from RBT events is needed. Currently, CTE can only be diagnosed post-mortem. Non-invasive, longitudinal neuroimaging studies may thus allow clinicians to visualize the underlying morphological, pathophysiological, and biochemical changes that occur in the acute stages of mTBI and chronic stages of RBT. Monitoring the progression of imaging signatures of RBT may provide insight into the underlying mechanisms of head trauma and provide imaging biomarkers to evaluate potential therapies.

Of further note, the distinction among different types of brain trauma informs the choice of the imaging modality to use to probe RBT. For example, in severe head trauma, where the structural damage is obvious, the most widely used neuroimaging techniques are computed tomography (CT) and conventional magnetic resonance imaging (MRI). On the other hand, findings on conventional neuroimaging are often insensitive to mild injury [11]; they are often non-specific to RBT or are only observable at late disease stages; that is, CTE. Further, imaging methods beyond morphological CT/MRI may be more sensitive to the pathological changes in RBT, such as shearing effects from blast injuries [12]. Such methods are being explored today and include advanced MRI techniques such as diffusion tensor imaging (DTI), susceptibility-weighted imaging (SWI), functional MRI (fMRI), magnetic resonance spectroscopy (MRS), and positron emission tomography (PET).

The purpose of this article is to provide an overview of current neuroimaging methods used to study RBT. While numerous reviews exist describing the use of neuroimaging in mTBI [11,13,14], few focus strictly on RBT. Therefore, a comprehensive literature review is provided here that focuses on RBT in human subjects. Studies were selected if: a) RBT was explicitly stated and it was clear that subjects had sustained more than one concussion; or b) subjects of the study were athletes and/or soldiers who are likely to experience RBT; or c) they were on subconcussive brain injury.

## Diffusion tensor magnetic resonance imaging

Animal and histological studies implicate microstructural injury as an early manifestation of traumatic brain injury (TBI) [7]. In particular, white matter (WM) tract injury is frequently observed in TBI often prior to macroscopic changes to brain tissue and before chronic manifestations of injury such as phosphorylated tau or amyloid deposition [15].

DTI is an analysis technique of non-invasive diffusion MRI that is sensitive to the diffusion of water within tissues. It has been mainly used to monitor WM tracts in the brain [16], since the microstructure of fiber tracts in WM presents a directional dependence to water diffusion within their vicinity, where the diffusion of water parallel to axons is (normally) faster than that in the perpendicular directions. Scalar measures, such as fractional anisotropy (FA), typically scaled between 0 and 1 are used to indicate the degree of directional dependence. Low values indicate isotropic diffusion; that is, equal in all directions, as would be observed in cerebrospinal fluid or gray matter (GM). High values indicate anisotropic diffusion; that is, diffusion that has a preferred orientation, as would be observed in WM where water displaces faster parallel to the fiber, bi-directionally [17]. Specialized diffusion MRI acquisitions that measure water diffusion in multiple directions combined with advanced processing methods convert the acquired signal into diffusion tensor maps and into various other scalar maps that provide additional information about the WM tracts in the brain. Common scalar metrics derived from the DTI analysis are shown in Table 1.

**Table 1:** Common scalar metrics for diffusion tensor imaging analysis

**Metric**	**Description**
Fractional anisotropy (FA)	Describes the anisotropy of water diffusion. Measured as a unitless scalar value between 0 and 1 with 0 being completely isotropic. Thought to be related to WM integrity. Changes in FA have been associated with TBI
Relative anisotropy (RA)	Represents the ratio of the anisotropic part of the diffusion tensor to its isotropic part. An alternative measure for FA
Axial diffusivity (AD)	Describes the magnitude of diffusion along its principal orientation. In WM this will measure diffusion along the axon. May be more specific to axonal degeneration
Radial diffusivity (RD)	Describes the magnitude of the diffusion in the plane perpendicular to the principal orientation. In WM, radial diffusivity is perpendicular to the fiber orientation. May be modulated by myelin in the WM
Mean diffusivity (MD)	Describes the magnitude of diffusion regardless of direction. Proportional to the trace of the diffusion tensor, with units of m^2^/s. Also known as the apparent diffusion coefficient (ADC). Increased MD has been associated with TBI

TBI, traumatic brain injury; WM, white matter.

### Diffusion tensor imaging changes in repetitive brain trauma

DTI has been extensively studied in severe TBI and mTBI. The reader is referred to recent excellent reviews that provide extensive coverage of these studies [11,18,19]. Here, we focus on studies that have explored DTI in the context of RBT.

Early DTI studies of patients with likely RBT focused on boxers. For example, Zhang and colleagues [20] performed anatomic MRI and DTI in boxers and matched controls. They found that both the mean diffusivity (MD) averaged over the whole brain (MD_av_) and its distribution width (σ) were significantly increased compared to controls. Increased MD_av_ was related to the frequency of hospitalizations for the boxers and may be present without any obvious anatomic anomalies. Chappell and colleagues [21] explored spatial heterogeneity of the diffusion signal in a cohort of boxers. Compared to controls, increases in MD and decreases in FA were identified in the WM, while decreases in MD were observed in GM. Interestingly, the authors found MD increases in the cerebellum, where defects in glucose metabolism in RBT patients have been seen with PET [22]. In a subsequent study, the authors found that a multivariate, linear discrimination approach was able to reveal more diffuse microstructure changes in the subcortical regions of the brain such as the thalamus and internal capsules in a cohort of boxers compared to traditional means of DTI analysis [23].

Recent studies have focused on RBT in athletes and war veterans. For example, MacDonald and colleagues [24] demonstrated decreased relative anisotropy in the cingulum, middle cerebellar peduncle, and right orbitofrontal WM in war veterans who had experienced blast injury. Increased radial diffusivity and MD were also observed, which could be indicative of axonal injury or edema or cellular inflammation. Follow-up scans 6 to 12 months later showed normalization of radial diffusivity and MD compared to controls, but decreased axial diffusivity and relative anisotropy. This is consistent with persistent injury with resolution of edema. However, such changes have not been consistent among studies. Specifically, Levin and colleagues [25] did not find FA and MD differences between veterans with blast injuries compared to controls, whereas a most recent study by Petri and colleagues [26] showed reduced FA in the corpus collosum, although it is of interest to note that they also do not show an effect of comorbid post-traumatic stress. With regards to sports-related RBT, Lipton and colleagues [27] found that the amount of ball heading in amateur soccer players was associated with lower FA in temporo-occipital WM, while Bazarian and colleagues [28] showed significantly increased FA and MD in high school athletes experiencing concussion throughout a sports season. Of note, Strain and colleagues [29] showed that frontal lobe FA is negatively correlated with measures of depression, highlighting the use of DTI as a biomarker of behavioral disturbance in RBT.

**Figure 1:**
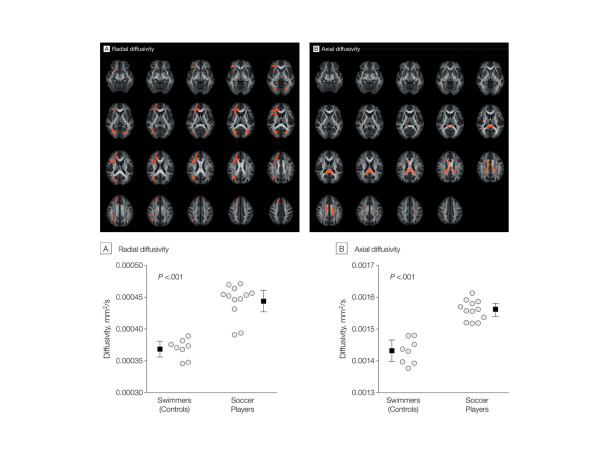
**Results of the Tract-based spatial statistics analysis and diffusivity measures for individual swimmers and soccer players.** Top: the diffusion tensor for each voxel was estimated by the multivariate linear fitting algorithm, and the tensor matrix was diagonalized to obtain three pairs of eigenvalues and eigenvectors. Voxelwise summary parameters included radial diffusivity and axial diffusivity. Group analyses were performed using whole-brain threshold-free cluster enhancement to obtain significant differences between groups at *P* < 0.05. After accounting for multiple comparisons using the family-wise error rate, the voxels highlighted in red demonstrate significantly increased radial diffusivity **(A)** and axial diffusivity **(B)** values for the soccer group compared with swimmers. Bottom: voxels with a significant group difference as revealed by Tract-based spatial statistics (top) were merged to a single cluster. Circles indicate individual values, squares indicate mean values, and error bars indicate 95% confidence intervals. Diffusivity measures were obtained for each individual and plotted for the two study groups. Linear regression showed no significant association of age or years of training with (A) radial diffusivity (*P* = 0.13 and *P* = 0.12, respectively) or (B) for axial diffusivity values (*P* = 0.22 and *P* = 0.54, respectively). Used with permission from [30].

Koerte and colleagues [30] recently examined WM alterations in active professional soccer players without a history of symptomatic concussion. Twelve male athletes trained for a career as professional soccer players since childhood at an elite level soccer club in Germany were compared to competitive swimmers. Tract-based spatial statistics revealed widespread differences between the two groups, with increased radial diffusivity in soccer players. Axial diffusivity was higher in the corpus callosum in the soccer players, as shown in Figure 1. Widespread diffusivity changes in soccer players compared with swimmers are similar to those observed in traumatic axonal injury following mTBI, suggesting that frequent subconcussive brain trauma even in the absence of a symptomatic concussion may affect WM microstructure. This first link between frequent subconcussive brain trauma and WM alterations was supported by a study on ice hockey players who showed increased diffusivity measures over the course of one play season [31]. Three of the investigated athletes sustained a concussion during the play season and were later found to have the most pronounced changes. Although there is evidence for WM alterations following repetitive subconcussive brain trauma, it is unknown if these findings represent initial evidence of neurodegeneration in CTE or are the direct, chronic effects of injury (for example, axonopathy).

Several factors need to be considered when interpreting DTI results. The sample sizes, especially controls, are often small. There is also often a wide inter- and intra-group variability in the RBT subjects and controls studied; that is, the severity of trauma in patients studied to date range from subconcussive episodes, to concussions, to mTBI and severe TBI, all of which can affect DTI results differently. MacDonald and colleagues [24] note that their subject recruitment method may have been biased towards the more severely injured. Cubon and colleagues [32] observed that MD may be more sensitive to mild injury while FA may be more sensitive to severe TBI. On the other hand, Lipton and colleagues [33] have reported increased FA early post-injury, which tends to predict good outcome. Additionally, latency between traumatic episodes and imaging may also affect results. This is especially highlighted in animal models of RBT, where the presence of significant findings on DTI has been found to be different at different time points after injury, thus showing a difference between acute and chronic injury [34,35]. Finally, the heterogeneity of DTI indices presenting in both control and RBT subjects needs to be considered. One solution is to build a normative atlas representing the reference ranges of DTI indices across the brain in a healthy population. A test subject’s diffusion measures are compared to the atlas and regions with a signal out of the normal range are flagged as abnormal (most commonly through z-scores). The resulting subject-specific profiles of injury can be summarized with location-independent measures such as ‘load’ (number of abnormal regions) or ‘severity’ (largest absolute z-score) and used for performing group comparisons [36].

### Summary

Studies to date have shown that DTI is sensitive to WM changes in both acute TBI and RBT. Future studies that delineate the time dependence of DTI changes due to RBT and the relationship between the frequency and magnitude of the trauma to DTI changes will provide more insight into conditions such as CTE [13]. Additionally, advanced diffusion MRI techniques may be more sensitive to microstructural changes than DTI [37,38]. Such advanced techniques typically require either high angular resolution (HARDI) or high radial resolution, or both. The HARDI acquisition measures multiple diffusion directions, and radial resolution can be obtained by acquiring the data in multiple diffusion sensitivities (b-values). In addition to greater sensitivity to microstructural changes, these additional measures also provide a better characterization of crossing fibers for tractography. As a result, these methods require longer acquisition schemes, which are less feasible in clinical setups. Nevertheless, with current advancement of hardware and acceleration methods, such acquisition schemes are expected to become clinically feasible in the foreseeable future [39]. Of special notice is the free-water imaging method, which can be applied retroactively on DTI data, and therefore does not require specialized acquisition [40]. The free-water method eliminates partial volume with water molecules that are free to diffuse in the extracellular space, providing better estimates of diffusivities within the tissue [41]. The output measures are the same as those provided by DTI but corrected for the partial volume effect and are thus more specific to changes in the tissue. In addition, the method provides an estimate of the volume of the extracellular free water, which appears to be indicative of pathologies such as atrophy and neuroinflammation [42]. Preliminary results on TBI patients show promise, since the method is able to distinguish between alterations that affect tissue versus those that affect the extracellular space [43]. These distinctions might be important to identify early stages of CTE in RBT patients. Finally, combining DTI results with other imaging information will likely also be most helpful in future studies [44].

## Magnetic resonance spectroscopy

MRS is a non-invasive technique that examines physiological metabolism *in vivo*. Using standard magnetic resonance scanners, chemical metabolites from tissue regions of interest are detected and shown as a spectrum depicting the type and concentration of the metabolites present. Localization of the signal can be from a single cubic volume (single voxel spectroscopy) or may utilize additional excitation pulses and scan time to provide information regarding spatial variations of these metabolites within a large region of interest (chemical shift imaging) [45]. The choice of echo time can influence which metabolites are detected based on their relaxation properties. Some MRS methods take advantage of this property to provide greater chemical specificity, such as spectral editing methods [46] or two-dimensional correlated spectroscopy (2D COSY), which obtains spectra at multiple echo times that, upon Fourier transform, provide spectral information in two dimensions (as opposed to spatial information in chemical shift imaging) [47]. Furthermore, MRS can detect the presence of metabolites via a variety of isotopes, such as ^1^H, phosphorus (^32^P), sodium (^23^Na) and carbon (^13^C). MRS has been demonstrated to be useful in multiple body systems, but its greatest application has been in the study of neurological disorders, including neuroinflammatory diseases, dementia and brain cancers. Typical metabolites relevant to brain studies using ^1^H MRS are summarized in Table 2.

**Table 2:** **Typical metabolites examined in neurological **^**1**^**H magnetic resonance spectroscopy [**[13]**]**

**Metabolite**	**Chemical shift resonance peak (ppm)**	**Comment**
Lipid	0.9-1.5	Not usually visible in MRS unless released by pathological process such as trauma
Lactate	1.3	End product of anaerobic glycolysis. Indicator of hypoxia and impairment of perfusion
N-acetyl aspartate (NAA)	2.0	Synthesized in neurons. Marker of neuronal viability
Glutamate/glutamine (Glx)	2.2-2.5	Glutamate is the primary excitatory neurotransmitter in the brain. Glutamine is found in astrocytes. Glx may be predictive of outcome after severe TBI and associated with immunoexcitotoxicity or secondary dysfunction
Choline (Cho)	3.2	Membrane marker. Elevated in cases of high membrane turnover. May indicate diffuse axonal injury
Myo-inositol (mI)	3.5	Astrocyte marker and osmolyte. Involved in the metabolism of phosphatidyl inositol (a membrane phospholipid) - expected to increase after TBI due to membrane damage. May also be a glial marker
Creatine (Cr)	3.0	Found in metabolic active tissues, used in energy storage and transfer. Used as an internal standard for other metabolites (for example, NAA/Cr, Cho/Cr)

MRS, magnetic resonance spectroscopy; TBI, traumatic brain injury.

The majority of MRS studies have examined metabolic changes after acute TBI events [48]. The following characteristic metabolic patterns have emerged from these studies to date as described in a recent review [14]. First, decreased N-acetyl aspartate (NAA (and NAA/creatine (Cr), NAA/choline (Cho)) levels are almost always observed after TBI in both WM and GM. This decrease can be present whether the injury is severe or mild and has been associated with diffuse axonal injury and neuronal loss. Second, increased Cho levels are also generally seen after injury. Third, elevated myo-inositol (mI), glutamine/glutamate (Glx) and lactate have also been observed. However, other studies have not shown these metabolic changes. The often high inter- and intra-variability between studies with regards to the characteristics of both patient and control cohorts, the mechanism of injury, the imaging time point post-injury, the MRS technique, and the location within the brain in which MRS was performed have made comparisons between studies difficult and further highlight the heterogeneity of the brain’s response to TBI. For example, Maugans and colleagues [49] demonstrated no differences in NAA between children aged 11 to 15 years after a single concussion compared to controls, suggesting that the pediatric brain may have neuroprotective mechanisms not present in adults. Chamard and colleagues [44] showed decreased mI/Cr in the motor cortex compared to controls in female athletes participating in multiple sports more than 7 months after a concussion. Female hockey players have also been observed to have a greater decrease in NAA/Cr compared to their male counterparts throughout the course of a season [50], suggesting that the impact of TBI on brain metabolism may be sex-dependent. Spatial heterogeneity of metabolites has also been noted. Yeo and colleagues [51] showed that Glx was increased in WM but decreased in GM compared to controls, while Govindaraju and colleagues [52] showed that NAA/Cho can differ significantly between different anatomical brain regions. Further studies are needed to explore the influence of these variables on brain metabolism in TBI.

Longitudinal studies have been performed to account for some of the confounding factors mentioned above and to understand the evolution of the brain’s response to TBI [49,51,53-55]. However, results from different studies remain mixed. Garnett and colleagues, for example, showed a decrease in NAA/Cr and NAA/Cho and increases in both Cho/Cr and mI/Cr in frontal WM within 1 week post-TBI compared to controls [56]. These changes were still present approximately 6 months later. NAA/Cr changes also correlated with clinical measures of outcome. Similarly, Henry and colleagues [53] observed decreased NAA/Cr in the prefrontal and motor cortices compared to controls in athletes 5 days after a concussive event. This decrease persisted 6 months later. An elevated mI/Cr was also seen in the motor cortex at the 6 month time point, suggesting the presence of increased numbers of glial cells. By comparison, Vagnozzi and colleagues [54] demonstrated a significant NAA/Cr and NAA/Cho decrease within frontal lobe WM in athletes within 3 days after a concussive event compared to controls, but no increase in Cho/Cr. NAA/Cr and NAA/Cho recovered by day 30 after injury [54]. Yeo and colleagues [51] observed increases in Cr and Glx in the WM and decreased Glx in the GM within 1 month of injury in patients compared to controls, with subsequent normalization to control values 3 to 5 months later. No changes in NAA values were seen. Overall, the temporal pattern of brain injury shows an initial decrease in NAA, reflective of neuronal injury that appears to be more evident in cortical GM brain regions, which generally recover to normal levels within 1 month. Changes in Glx and mI, tied to excitoxicity and glial cell proliferation, respectively, appear to be more long-standing. It is important to note that both Glx and mI are only observed using short-echo spectroscopy, which is the reason why other studies utilizing long-echo methods did not detect these changes. Changes in Cho levels appear to be more variable. This may be dependent on the type and extent of brain injury as Cho is related to membrane turnover or diffuse axonal injury.

### Magnetic resonance spectroscopy changes in repetitive brain trauma

Several studies have examined brain metabolism using MRS in subjects with likely RBT. Tremblay and colleagues [57] used MRS to examine former ice hockey and football players aged 51 to 75 years with multiple concussions. Along with ventricular enlargement and cortical thinning, they found elevated mI in the left medial temporal lobe along with increased Cho in the prefrontal cortex. The mI changes correlated with episodic memory decline. In another study, Davie and colleagues [58] examined three ex-professional boxers with parkinsonian syndrome. NAA was found to be significantly decreased in the lentiform nucleus in these subjects compared to matched controls and idiopathic Parkinson’s disease patients. This study implicated neuronal loss due to post-traumatic encephalopathy for the boxers’ clinical symptoms, but NAA changes due to parkinsonism cannot be ruled out [59]. A recent study by Hetherington and colleagues [60] demonstrated decreased hippocampal NAA/Cr and NAA/Cho in Iraq and Afghanistan war veterans who experienced multiple blast injuries with memory impairment compared to controls. This study is unique in demonstrating the feasibility of acquiring MRS data on a 7 T MRI system. Vagnozzi and colleagues [55] demonstrated that RBT can prolong the recovery of NAA after a TBI event. Athletes who experienced repeated concussion within 2 weeks of the original TBI continued to have depressed NAA/Cr 30 days after the initial trauma whereas singly concussed subjects returned to control levels of NAA/Cr by that time. A study by the same group in an animal model of RBT demonstrated that multiple mild traumatic episodes experienced over short time intervals can depress brain NAA levels (measured using high-performance liquid chromatography of brain extracts) to levels lower than a single severe TBI event. These results corresponded with lower ATP and ADP in the brain [61] and are concordant with glucose metabolism changes observed in a RBT model [62]. Taken together, these results suggest that TBI may result in a prolonged period of brain vulnerability to further injury. RBT within this vulnerable period, however mild, may result in injury comparable to that seen in severe TBI.

**Figure 2:**
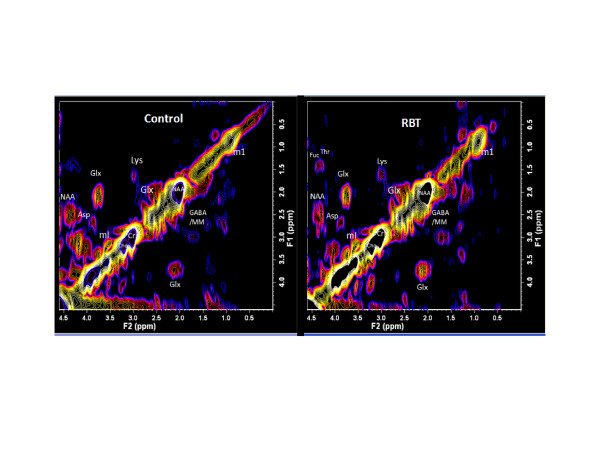
**L-COSY spectra from healthy control (left) and athlete with a history of repetitive brain trauma (RBT; right).** Spectroscopy was performed at 3T using a 32 channel head coil and voxel size of 3 × 3 × 3 cm^3^ in the posterior cingulated gyrus; increment size 0.8 ms; 64 increments with 8 averages resulting in an acquisition time of 12.8 minutes; acquired vector 1,024 points; acquisition time 512 ms; spectral width in F2 2,000 Hz and spectral width in F1 1,250 Hz. For presentation the spectra were calibrated to the lysine cross peak at 3.00 to 1.67 ppm. Asp, aspartate; Cho, choline; Cr, creatine; Fuc, fucose; GABA, gamma-aminobutyric acid; Glx, glutamate/glutamine; Lys, lysine; m1, macromolecule; mI, myo-insitol; NAA, N-acetyl aspartate; Thr, threonine.

Many metabolites are measureable in the human brain by MRS, but in conventional MRS many of the resonances overlap, even at 3T, making it difficult to differentiate individual metabolites. Using 2D COSY, J-coupling between protons in molecules results in cross-peaks that allow for unambiguous identification of up to 35 different metabolites [63,64]. In a pilot study (Lin AP, Ramadan S, Box H, Stanwell P, Stern R, unpublished data), 2D COSY showed additional neurochemical changes in this athlete cohort not previously observed by MRS in brain injury or neurodegenerative disease, such as changes in aspartate, threonine, and glutathione. A representative 2D COSY from a former NFL player is shown in Figure 2. In addition, results also show increased Cho and Glx in athletes compared with controls, which were statistically significant despite the small sample size. Increased Cho and Glx are consistent with diffuse axonal injury and excitotoxic injury. Of particular interest is an observed increase in mI in professional football players with RBT. mI has been reported by others as an early diagnostic marker for mild cognitive impairment [65], is also increased in those with axial diffusivity [66,67], and has been shown in mouse models to be directly related to the presence of phosphorylated tau [68,69].

### Summary

MRS studies to date demonstrate that brain metabolic derangements are present in both acute TBI and RBT. MRS has been shown to be sensitive to these changes. Improvement in MRS techniques that can increase signal to noise, provide robust, high quality spectra [60], and that resolve closely associated metabolite peaks [70] may allow improved quantification of the metabolites currently being studied as well as the discovery of other metabolites relevant to RBT. Further studies with isotopes other than ^1^H are also warranted [71]. It is important to note that most studies discussed here measure metabolite ratios, most often in relation to Cr. Although Cr is assumed to be generally unchanged in the normal brain, this may not be the case after TBI [51]. Changes in NAA/Cho may be a useful clinical biomarker of RBT prognosis and treatment response, but its ability to explain the mechanism behind the changes, given that both NAA and Cho are hypothesized to change after a TBI, is also unclear.

As discussed above, carefully planned future clinical studies to minimize confounding factors are needed to clarify the significance of each metabolite biomarker during the course of RBT. In particular, careful choice of MRS acquisition parameters is essential. Also, matched controls to RBT subjects are important for comparison in RBT and sports-related injuries. Chamard and colleagues [44] noted female athletes ‘not clinically identified as sustaining a concussion’ showed decreases in NAA/Cr. Thus, subconcussive blows experienced during the regular course of play or training may need to be considered as a factor in future analyses of sports-related RBT. Correlation of clinical MRS results with animal studies of RBT as well as with studies using other modalities such as nuclear imaging, structural MRI [57], fMRI [72] and DTI will also aid in interpreting future MRS findings.

## Functional magnetic resonance imaging

Since first demonstrated in humans in 1992, fMRI has revolutionized neuroscience. It is used as a research tool in brain mapping and connectivity studies, as well as in the clinic for surgical planning and treatment response. The specific contrast in fMRI is based on the blood oxygen level dependent (BOLD) contrast mechanism that stems from the presence of deoxyhemoglobin. The assumption made in BOLD-fMRI is that there is a coupling between neuronal activity within a brain region and a local increase in cerebral blood flow. Thus, BOLD-fMRI is likely reflective of the hemodynamic response to neuronal firing [73].

Few studies have been performed to examine mTBI using fMRI, the majority of them since 2009. McDonald and colleagues [74] provide a comprehensive review of existing fMRI studies, noting that most have focused on executive function, working memory and episodic memory performance. Resting state fMRI, which can probe intrinsic connectivity of different brain regions without task performance, has also been applied to mTBI [75]. To date, most studies demonstrate differences in BOLD-activation between mTBI patients and controls. Enhanced BOLD signal has been observed in the prefrontal and dorsolateral prefrontal cortex while performing cognitive tasks in mTBI patients [73]. However, hypoactivation after injury has also been observed in both clinical [76] and preclinical [77] studies. The majority of studies focus on the subacute stage of injury and in relatively young populations. Inconsistencies may result from individual differences and methodologies (in both tasks and post-processing). Future studies examining longitudinal changes and in factors such as aging and comorbid conditions are necessary to help establish the value of this method.

### Functional magnetic resonance imaging and repetitive brain trauma

A subset of fMRI studies has examined populations with likely RBT. For example, in a study by Scheibel and colleagues [78] brain activation was observed in 15 soldiers with blast injuries (all male, 11 with multiple blasts exposures, 6 with multiple blast-related TBIs, imaged on average 2.6 years post-injury) who served in Iraq and Afghanistan. Compared to controls, soldiers with TBI showed increased activation in the anterior cingulate gyrus, medial frontal cortex and posterior cerebral areas. No differences in the fMRI task accuracy were seen between cohorts, although the blast group showed slower response times. Activation was negatively correlated with symptoms of post-traumatic stress disorder (PTSD). Matthews and colleagues [79] examined soldiers with loss of or altered consciousness after multiple blast-related injuries with stop task fMRI. Although there were no differences in task performance between the groups, loss-of-consciousness patients showed decreased activation in the left ventromedial prefrontal cortex during easy trials, which positively correlated with somatic symptom severity. Since the ventromedial prefrontal cortex has been thought to be involved in self-awareness, the authors interpreted the results as suggesting that loss-of-consciousness patients were less self-aware, and thus reported fewer somatic symptoms. This finding, however, while intriguing, needs to be followed up in future studies.

Talavage and colleagues [80,81] have used longitudinal fMRI to study high school football players with RBT during multiple football seasons. Along with players who showed both clinical and fMRI alterations after concussion (clinically observed impairment (COI)+/functionally observed impairment (FOI)+), they identified a subset of players who did not show clinical symptoms of head injury but presented with alterations on fMRI compared to baseline at the beginning of the season (COI-/FOI+). COI+/FOI + subjects showed increased activations particularly in the posterior middle and superior temporal gyri while COI-/FOI + subjects showed increased activations in the dorsolateral frontal cortex, cerebellum and upper parietal and occipital regions. These findings were consistent with deficits in neurocognitive testing, which showed verbal working memory deficits in COI+/FOI + individuals compared to impaired visual working memory in COI-/FOI + subjects. Interestingly, COI-/FOI + individuals experienced more high impact collision events (>20 G) to the head compared to both COI-/FOI- and COI+/FOI + cohorts. These studies support the assertion that the pathophysiology due to acute TBI and RBT may be quite different.

### Summary

fMRI has demonstrated neural activation differences between individuals with TBI and controls. Unique fMRI changes in subjects with subconcussive RBT have also been observed. Further studies are needed to validate these findings. The ability to acquire longitudinal functional information in a single subject with fMRI, without the need for ionizing radiation (for example, PET), will also enable the monitoring of long-term effects of RBT and potential treatments for TBI or CTE [77]. It is especially important for future studies to determine the neurological mechanism of these fMRI alterations.

## Susceptibility-weighted imaging

SWI is a MRI technique explored for its sensitivity to micro-hemorrhage [82]. The presence of blood breakdown products such as hemosiderin and ferritin, and deoxyhemoglobin in blood can distort the local magnetic field, causing changes in local tissue susceptibility that are observable with gradient-echo (GRE) MRI. SWI is based on the observation that the phase component of GRE data contains substantial information about such local tissue susceptibilities. In SWI, phase information from flow-compensated GRE data is processed, filtered and combined with magnitude information to provide images with enhanced contrast information compared to conventional MRI. SWI is more sensitive to micro-bleeds than conventional GRE [83]. The technique has been applied to multiple conditions, including stroke, vascular disease and the visualization of micro-bleeds in TBI [84].

Scheid and colleagues [85] found a high frequency of micro-bleeds in the frontal, parietal, and temporal lobes using GRE sequences in patients with chronic (mean of 2 years post-injury) mTBI to severe TBI. The number of micro-bleeds correlated with the presence of brain atrophy, callosal lesions and Glasgow Coma Scale but not with the Glasgow Outcome Scale [85]. SWI studies in pediatric populations have demonstrated good correlation between TBI severity and the number of hemorrhagic lesions visualized [86,87]. High frequency lesion regions include the frontal WM and the parieto-temporal-occipital regions. Increased numbers of lesions may be associated with poor neuropsychological outcome [88]. However, Toth and colleagues [89] did not observe micro-hemorrhages using SWI in adult patients with acute and subacute mTBI compared to controls, even though DTI demonstrated significant changes in MD and FA. More studies are thus needed to determine under what circumstances micro-hemorrhages are observed and are associated with neurocognitive symptoms.

### Susceptibility-weighted imaging and repetitive brain trauma

Breakdown of the blood–brain barrier, changes in the cerebral vasculature and perivascular deposition of tau are also hypothesized to occur in CTE [13]. Thus, SWI could potentially be a useful biomarker for RBT. However, very few studies have used SWI to detect micro-bleeds in RBT, with the exception of two studies in boxers. In the first study, Hahnel and colleagues [90] found 3 out of 42 boxers showed micro-hemorrhages with SWI, while in the second study Hasiloglu and colleagues [91] found micro-hemorrhages in 2 out of 21 boxers. While no hemorrhages were seen in controls in either of these studies, the differences in prevalence of lesions between boxers and controls were not significant. Of note, these studies were conducted at 1.5 T, where susceptibility is not as evident. Therefore, further studies are necessary to assess the utility of SWI in RBT.

### Summary

Studies using high-field MRI (>3.0 T) will enhance SWI contrast [92] due to increased susceptibility at higher field. However, standardization of SWI processing is necessary to compare results between studies. In addition, biomarkers other than micro-hemorrhage, such as oxygen saturation or venous changes, may also be examined with SWI [93]. As with other modalities, the SWI signal will be time course-dependent [94]. So far there have been no longitudinal studies of RBT using SWI. As SWI is an emerging technology, future studies will determine the efficacy of this method for RBT.

## Positron emission tomography

PET is a nuclear imaging technique that has several advantages compared to other nuclear imaging techniques such as single-photon emission computed tomography [95]. It is highly sensitive, requiring tracer amounts of a radio-nuclide for image formation. The high sensitivity also allows for relatively short scan times, important for dynamic PET studies and in the clinical setting. Moreover, positron emitting isotopes include carbon, nitrogen, oxygen and fluorine; these are found in many biological compounds of interest and can be readily incorporated into radiopharmaceutical analogs for imaging of physiological function. Finally, in the context of RBT, PET is a quantitative technique, enabling longitudinal studies on the same subject to be performed. However, these benefits are tempered by the relatively high cost of PET and concerns about elevated ionizing radiation exposure to the patient.

### Metabolic changes during brain injury with positron emission tomography

Most studies of TBI involving PET seek to evaluate changes to the brain’s glucose metabolism post-trauma using 2-deoxy-2-(^18^F)-fluoro-D-glucose (FDG). FDG is an analog of glucose that is taken up by cells with high glucose metabolism such as in the brain, cancer and in areas of inflammation. FDG is trapped within cells after uptake and does not complete glycolysis, enabling it to provide PET images depicting areas of high glycolytic activity.

Most FDG-PET studies to date have evaluated brain metabolism after acute TBI. These studies demonstrated abnormal patterns of the cerebral metabolic rate of glucose (CMRglc) months to years after the injury [96-98]. However, the small sample sizes and differences in the subject population, type of injury experienced [99], PET acquisition protocols and the time duration between the injury event and imaging make it difficult to draw solid conclusions from these studies. In general, FDG studies performed in a resting state [97,98] or with performance stimuli [98,100] all demonstrate regions of glucose hypometabolism. Hypometabolism was observed in most studies within the frontal and temporal regions and correlated with neuropsychological testing, but not with structural defects seen with MRI or CT. Regions of hypermetabolism have also been observed in some studies [98,100]. Differences in the spatio-temporal patterns of CMRglc observed in the FDG-PET studies may be partially explained by individual rates of metabolic recovery after the TBI event [101,102].

Recent FDG-PET studies have also examined glucose metabolism in subjects with a high likelihood of RBT. Provenzano and colleagues compared FDG uptake patterns between professional and amateur boxers with controls [103]. They showed an 8 to 15% decrease of FDG uptake within the posterior cingulate cortex, parieto-occipito, frontal lobes bilaterally and the cerebellum in the boxers compared to controls, claiming that this represents a unique pattern of hypometabolism associated with chronic traumatic brain injury in boxers. However, the fact that some of these regions of hypometabolism have been observed in previous studies of single-event TBI in admittedly heterogeneous patient cohorts makes this claim difficult to validate at this time. In a study that examined FDG uptake in Iraq war veterans with multiple (3 to 51) blast exposures, Peskind and colleagues [22] reported hypometabolism in the medial temporal lobes, cerebellum, vermis and pons. Confounding factors in this study included the fact that controls were not matched for age or occupation and the presence of PTSD in 10 of the 12 subjects studied. However, it is interesting to note that previous studies of PTSD patients did not show hypometabolism in the cerebellum, as was observed by Bremner and colleagues [104] and Petrie and colleagues [26] who reported that PTSD was not associated with a comorbid effect in veterans with blast injury but was associated with reduced cerebral glucose metabolism in the parietal, somatosensory, and visual cortices when comparing veterans with and without blast or impact injury. To account for the latter confound, Mendez and colleagues [105] studied war veterans in whom PTSD had been excluded. Further, they examined differences in FDG metabolism between those with repetitive blast injuries compared to blunt injuries. Blast injuries are hypothesized to be more severe due to the presence of additional trauma secondary to the initial impact. Compared to controls, hypometabolism was noted for both blast and blunt injury groups in multiple regions, including the left frontal and temporal regions as well as the thalamus, while hypermetabolism was noted in the right caudate and temporal regions. Interestingly, subjects with blast injury demonstrated significant hypometabolism in the right superior parietal region compared to those who experienced blunt injury. Rather than a focal injury, the authors suggest that this may be sequelae of diffuse structural damage.

While these studies demonstrate that abnormal deviations of glucose metabolism are characteristic of both TBI and RBT, the spatio-temporal patterns of these deviations remain inconsistent between studies. Future studies that reduce confounding between subjects, data acquisition and analysis are warranted. Chen and colleagues [99] suggest that PET imaging during a working memory task using H_2_[^15^O] may be a more sensitive biomarker than FDG-PET for mTBI. Further, animal studies may offer insight into the human results. For example, Prins and colleagues [62] demonstrated in a rat model of RBT that temporal latency between traumatic events can significantly affect CMRglc.

### Monitoring structural changes in repetitive brain trauma with positron emission tomography

Recent neuropathological studies of subjects with a history of RBT and CTE have identified aggregation and accumulation of hyperphosphorylated tau and TDP-43 as pathognomonic for CTE [13]. The ability to evaluate these proteins *in vivo* may offer a unique biomarker to diagnose CTE and understand the evolution of the disease. In a preliminary study, Small and colleagues [106] used 2-(1-(6-[(2-[^18^F] fluoroethyl)(methyl)amino]-2-naphthyl) ethylidene) malononitrile (FDDNP) for PET imaging in five retired National Football League players with a history of cognitive and mood symptoms. FDDNP binds to both tau neurofibrillary tangles and amyloid plaque in brain tissue [107]. Compared to matched controls, the football players showed increased FDDNP uptake in the caudate, putamen, thalamus, subthalamus, midbrain, cerebellum and amygdala. Interestingly, increased levels of uptake were associated with increased number of concussions experienced.

**Figure 3:**
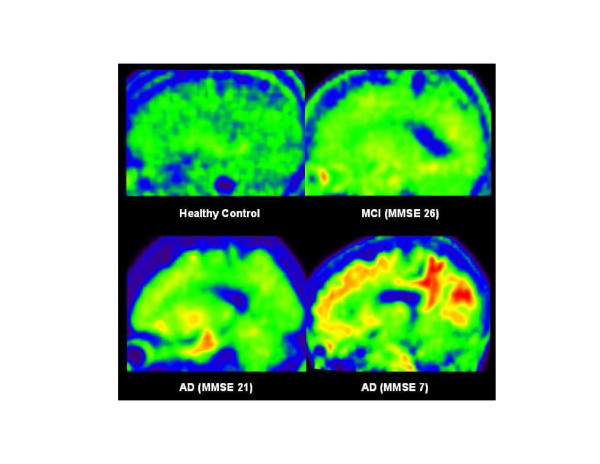
**T807 tau tracer.** Sagittal images from 80 to 100 minutes post-injection of a 56-year-old healthy subject (top left), mild cognitively impaired (MCI) subject (top right), mild Alzheimer’s disease (AD) subject with mini-mental state exam (MMSE) 21 (bottom left), and severe AD subject with MMSE 7 (bottom right). The intensity and extension of T807 uptake correlated to Braak and Braak stages of phosphorylated tau deposition, except in the area where severe neuronal degeneration is expected, for which the mild AD subject had the highest cortical retention. Reprinted from the *Journal of Alzheimer's Disease*, volume 34 (No 2) by Chien *et al*. Early Clinical PET Imaging Results with the Novel PHF-Tau Radioligand [F-18]-T807, p465, Copyright 2013, with permission from IOS Press [111].

While the study is interesting, it is based on a very small sample, and it is not obvious that FDDNP binding in regions of the brain that show tau deposition at autopsy in NFL players necessarily implies tau deposition in this study as FDDNP is not specific for tauopathies. There is great interest in developing a tau-specific ligand, particularly to investigate *in vivo* tau in NFL players in whom tau deposition, and not neuritic plaques, has been observed at autopsy [7]. PET probes that are specific for tau will be important in the context of RBT and CTE, and there are now several promising probes with good tau specificity that have been developed [108-111] and are being incorporated into *in vivo* imaging studies as shown in Figure 3.

### Neuroinflammation imaging with positron emission tomography

An associated sequelae of TBI is the brain’s neuroinflammatory response to injury. Glial tangles and inclusions have been noted in CTE. The peripheral benzodiazepine receptor (PBR) is found on primary activated microglia and phagocytic cells in the central nervous system [112]. Several groups have developed radiolabelled probes targeting the PBR as a means to evaluate neuroinflammation response in TBI. Folkersma and colleagues [113] showed increased binding of the PBR target (R)-^11^C-PK11195 across the whole brain in patients 6 months post-injury. A concurrent animal study by the same group correlated (R)-^11^C -PK11195 uptake with histological markers of microglia and brain injury [114]. In another study, Ramlackhansingh and colleagues [115] demonstrated (R)-^11^C -PK11195 binding up to 17 years post-TBI event, suggesting that chronic neuroinflammation can persist in the context of brain trauma. While (R)-^11^C -PK11195 is a promising probe that can localize activated microglia, its low binding specificity *in vivo* can reduce signal to noise of the images and complicate quantification of its uptake [116]. Novel methods are nonetheless being developed to analyze such PET data [117]. Concurrently, alternative probes with improved binding specificity are also being developed [118].

### Summary

The ability of PET to provide highly sensitive, quantitative and non-invasive images makes it ideal for studying RBT. Multiple PET studies have demonstrated changes in glucose metabolism, tau protein build up and neuroinflammation in the context of brain trauma. Future studies involving an increased number of subjects from multiple time points relative to traumatic events will validate the utility of the different PET biomarkers to evaluate RBT. Further, correlation of PET biomarkers with other imaging biomarkers, such as DTI [26] and MRS, will be extremely useful towards gaining a more comprehensive understanding of RBT.

## Conclusion

Research into RBT and CTE is still very much in its infancy, as many questions remain to be answered. Given that currently CTE can only be diagnosed post-mortem, it is imperative to identify *in vivo* biomarkers for CTE. The availability of such biomarkers will provide a platform on which treatments for this condition can be developed and evaluated.

As reviewed here, non-invasive neuroimaging studies show great promise in providing key imaging biomarkers to monitor CTE: DTI measures reveal WM changes that are reflective of diffuse axonal injury and other processes such as neurodegeneration. Similarly, MRS results are also reflective of diffuse axonal injury and neurodegeneration as well as providing insight into underlying pathophysiological processes such as disturbances in glutamatergic neurotransmission. fMRI methods also reveal insight into the brain activity by demonstrating different activation patterns in subjects with RBT. Micro-hemorrhages on SWI may provide additional morphological changes not seen using conventional imaging methods. Finally, PET imaging, particularly using tau-specific ligands, promise the most direct means of assessing CTE in RBT. While each of these methods show promise in providing diagnostic and potentially prognostic information, it is likely that a combination of these different imaging methods will provide a more complete picture of pathophysiological changes that are associated with the long-term effects of RBT.

However, challenges remain before these biomarkers can be translated to routine clinical use. The biggest challenge is the identification of imaging signatures that can parse the difference between acute brain injury, chronic effects of RBT, and the development of CTE. Imaging biomarkers that are specific to each of these conditions will be important for diagnosis, treatment, and hopefully prevention of progressive neurological damage. A number of factors need to be considered in the quest to identify these biomarkers. RBT by nature can be very heterogeneous; trauma to different parts of the brain via different mechanisms of trauma can result in different clinical presentations of brain injury. These different presentations may or may not share the same underlying pathophysiology. Genetic and environmental variations between individual patients likely also influence the imaging signatures. The studies cited above have already highlighted imaging differences in the neurological response to RBT between the sexes and between pediatric and adult populations. Apart from this, comorbidity of different diseases such as Alzheimer’s disease, PTSD, and/or depression may obfuscate the presentation of TBI or CTE. Furthermore, few current studies have characterized the longitudinal changes that occur in each of the different modalities nor have they determined whether or not neuroimaging biomarkers will be effective for treatment monitoring. Finally, in addition to examining the strength of multimodal imaging, the incorporation of neuroimaging results in overall metrics for RBT, including neuropsychological evaluation, blood and/or cerebrospinal fluid biomarkers, genetic tests (such as APOE), and clinical evaluation, will likely provide the most complete picture of the long-term effects of RBT.

## Note

This article is part of a series on *Traumatic brain injury*, edited by Robert Stern. Other articles in this series can be found at http://alzres.com/series/traumaticbraininjury

## Abbreviations

BOLD: Blood oxygen level dependent; Cho: Choline; CMRglc: Cerebral metabolic rate of glucose; COI: Clinically observed impairment; COSY: Correlated spectroscopy; Cr: Creatine; CT: Computed tomography; CTE: Chronic traumatic encephalopathy; DTI: Diffusion tensor imaging; FA: Fractional anisotropy; FDDNP: 2-(1-)6-[(2-[^18^F] fluoroethyl)(methyl)amino]-2-naphthyl) ethylidene) malononitrile; FDG: 2-deoxy-2-(^18^F)-fluoro-D-glucose; fMRI: Functional magnetic resonance imaging; FOI: Functionally observed impairment; Glx: Glutamine/glutamate; GM: Gray matter; GRE: Gradient echo; HARDI: High angular resolution; MD: Mean diffusivity; mI: Myo-inositol; MRI: Magnetic resonance imaging; MRS: Magnetic resonance spectroscopy; mTBI: Mild traumatic brain injury; NAA: N-acetyl aspartate; PBR: Peripheral benzodiazepine receptor; PET: Positron emission tomography; PTSD: Post-traumatic stress disorder; RBT: Repetitive brain trauma; SWI: Susceptibility-weighted imaging; TBI: Traumatic brain injury; WM: White matter.

## Competing interests

APL is a co-inventor of a patent entitled ‘Magnetic Resonance Spectroscopy Provides a Non Invasive Means of Monitoring Repetitive Head Injury’ (USPTO, ed. A61B5/055 ed. USA: Brigham and Women’s Hospital, 2011; US2011/062211). All other authors declare that they have no competing interests.

## Authors’ contributions

All authors contributed to and reviewed the manuscript. All authors read and approved the final manuscript.
